# Enhanced Text Spacing Improves Reading Performance in Individuals with Macular Disease

**DOI:** 10.1371/journal.pone.0080325

**Published:** 2013-11-11

**Authors:** Sally Blackmore-Wright, Mark A. Georgeson, Stephen J. Anderson

**Affiliations:** Neurosciences, School of Life & Health Sciences, Aston University, Birmingham, United Kingdom; New York University, United States of America

## Abstract

The search by many investigators for a solution to the reading problems encountered by individuals with no central vision has been long and, to date, not very fruitful. Most textual manipulations, including font size, have led to only modest gains in reading speed. Previous work on spatial integrative properties of peripheral retina suggests that ‘visual crowding’ may be a major factor contributing to inefficient reading. Crowding refers to the fact that juxtaposed targets viewed eccentrically may be difficult to identify. The purpose of this study was to assess the combined effects of line spacing and word spacing on the ability of individuals with age-related macular degeneration (ARMD) to read short passages of text that were printed with either high (87.5%) or low contrast (17.5%) letters. Low contrast text was used to avoid potential ceiling effects and to mimic a possible reduction in letter contrast with light scatter from media opacities. For both low and high contrast text, the fastest reading speeds we measured were for passages of text with double line and double word spacing. In comparison with standard single spacing, double word/line spacing increased reading speed by approximately 26% with high contrast text (p < 0.001), and by 46% with low contrast text (p < 0.001). In addition, double line/word spacing more than halved the number of reading errors obtained with single spaced text. We compare our results with previous reading studies on ARMD patients, and conclude that crowding is detrimental to reading and that its effects can be reduced with enhanced text spacing. Spacing is particularly important when the contrast of the text is reduced, as may occur with intraocular light scatter or poor viewing conditions. We recommend that macular disease patients should employ double line spacing and double-character word spacing to maximize their reading efficiency.

## Introduction

Macular disease is the leading cause of blindness in people over sixty years of age in many developed countries, and the third most common cause globally after cataract and glaucoma [1]. Central visual loss, as exhibited in macular disease, affects many aspects of life including the recognition of faces and facial expressions, watching television, cooking, driving, and reading – factors that impede a person’s ability to communicate, their leisure activities, and independence.

Because reading provides information, pleasure and a degree of independence not matched by many activities, there have been numerous attempts to improve the legibility of text by changing its style [2-4], size [5,6], font and line width [7,8], polarity [9], spatial frequency content [10,11], and colour [12]. Other factors that may be important for visually-disabled persons include text illumination [13,14], presentation method [15,16], retinal area used [17-21], oculomotor control [22,23], and perceptual training [24-27]. Although these changes all have some impact on letter and/or word acuity, the gains in reading speed are often modest [2]. In consequence, there remains no clear consensus on the optimal text parameters for reading without central vision.

The usual modification made to reading materials for the visually impaired is to make the print larger. This is grounded in the belief that peripherally-viewed text may be read more effectively if it is scaled to counter the coarse sampling evident in human peripheral retina [6,28]. Sampling issues aside, however, there are other differences in neural processing between central and peripheral vision that may have deleterious effects on reading, of which a prime example is the phenomenon of ‘crowding’. Crowding refers to the fact that a target (e.g. letter or word) in the peripheral visual field is much harder to identify in the presence of nearby targets [29-34]. Crowding is indicative that visual information is pooled over large retinal distances [31,35,36], and it may be a major factor contributing to inefficient reading with peripheral vision [27,37]. 

Several authors have investigated the effects of crowding by examining how letter spacing, vertical word spacing or horizontal word spacing affect reading performance. The results on letter spacing were unequivocal: all authors reported that increased letter spacing does not lead to an increase in reading speed [38-42]. This may be so because any advantage gained by minimizing the effects of letter crowding were negated by a reduction in word shape information and/or visual span, factors known to be critical for efficient reading [34,38,43]. The effects of vertical word spacing were mixed. In single word recognition tasks with eccentric viewing, increased vertical word spacing improved reading speed in normally-sighted individuals [29] but not in individuals with macular disease [44]. With whole sentences, enhanced interline spacing was reported to yield either a small increase in the maximal reading speed of macular disease patients [45] or no change at all [44]. There is indirect evidence that enhanced horizontal word spacing may also have a small positive effect on reading performance. For example, several studies have reported a reduction in reading rate (for English text) when interword spaces are reduced or removed [46-48], while increased interword spacing results in shorter average fixation durations [49-51]. The possible interactive effects of vertical and horizontal word spacing on reading performance are unknown.

The aim of this study was to assess the combined effects of line and word spacing on reading speed (number of correctly-read words per minute) in adults with binocular macular disease. We used whole sentences and allowed saccadic eye movements and binocular vision in order to model as closely as possible the normal reading process. 

## Methods

### Participants

We studied 24 participants with binocular macular degeneration, all selected from the outpatient eye clinic in the Gloucestershire Royal Hospital NHS Foundation Trust. Our inclusion criteria were: binocular macular degeneration (wet or dry) of any duration in adults (male or female) aged 18 yrs and over; binocular distance acuity between 0.3 and 0.8 logMAR; and English as primary language, with fluent reading abilities prior to central vision loss. Our exclusion criteria were: ocular co-morbidity; amblyopia; dyslexia; and any cognitive impairment.

### Critical print size

All participants who met our inclusion criteria were further screened according to their critical print size to ensure their suitability for the full study. Following a full optometric examination and near refractive correction for a viewing distance of 40 cm, critical print size was measured using Minnesota Low-vision Reading Test (MNREAD) charts. These are continuous-text reading acuity charts designed to measure reading speed in low vision patients [52-54]. They consist of 19 short sentences (60 characters, including spaces), each printed as three lines of left/right justified text in a proportionally spaced Times Roman font with single line/word spacing. The difficulty of the test increases as the observer reads down the chart, with each successive sentence being 0.1 logMAR smaller than the previous. At 40 cm, print size varied from 1.3 to -0.5 logMAR. In accordance with standard protocol, the time taken to read each sentence was measured to the nearest 0.1 s. With each sentence having the same cognitive load, number of characters and spatial layout, it is assumed that any change to reading speed between successive sentences is primarily due to the change in print size [52]. 

Printed with black letters on a white background, the measured Michelson contrast of our standard MNREAD chart was 87.5%. As the main experiment required an assessment of reading speed for both high and low contrast text (see below), we measured critical print size using both the standard MNREAD chart and a low contrast (17.5%) version of the chart. The low contrast version was developed by us specifically for use in this study. In both cases, the white sections of the cards were presented at a luminance of 100cd/m^2^, produced by both fluorescent overhead room lighting and a ‘daylight’ angle poise lamp. All measurements were performed binocularly at a viewing distance of 40 cm. 

Critical print size was measured as the smallest print size that supported a reading speed of at least 80% of the participant’s maximal reading speed, where the latter was defined as the single fastest reading speed across the range of print sizes [55,56]. Only participants with a critical print size no higher than 0.8 logMAR (N20) for both low (17.5%) and high contrast (87.5%) charts were selected to take part in the full study. This print size ensured that, even with maximal text spacing, the number of words per line was similar to normal reading materials. 

### Experimental test reading cards

Short passages of text describing simple aspects of marine life were written by the authors and used to assess reading performance in individuals with macular disease. Each passage comprised three sentences, with 51 words in total, including three complex words. A complex word was defined as one with three or more syllables, excluding common suffixes (-es, -ed or –ing) and proper nouns. Each passage was printed as black-on-white text on an A4-sized card using a Times New Roman font of size N24. This text size was sufficiently large to support each participant’s critical print size, but small enough to allow the text passage with maximal (i.e. triple) word and line spacing to be printed on a single card. To allow for all permutations of word and line spacing used (i.e. single, double and triple spacing), nine different passages were developed. Word spacing was varied using standard character (space-bar) spacing, while line spacing was varied in multiples of the height of an upper-case ‘X’. The readability of each passage was manipulated to achieve a Gunning fog index of 9.1, indicating that the text could be understood by someone who left full-time education at or after 9.1 years [57]. A duplicate set of cards were produced, one with a Michelson letter contrast of 87.5% and the other with a letter contrast of 17.5%, matching the measured contrasts of the standard and non-standard MNREAD charts used to determine critical print size. Note that the low contrast cards were used in an attempt to avoid any potential ceiling effects, and also to mimic a possible reduction in letter contrast with light scatter from media opacities.

### Procedure

For each reading time measure, the printed test cards were placed on a self-supporting stand at a viewing distance of 40 cm. A blank card covered the test card prior to each measure. Each participant, optically corrected for 40 cm, was asked to read aloud the paragraph on the test card as quickly as possible without making errors. Should an error be made, they were instructed to continue reading to the end of the paragraph. The examiner (SB-W) uncovered the test card and said ‘go’, starting the stop watch immediately and stopping it after the participant read the last word of the last sentence. Reading speed was computed as the number of correctly-read words per minute (wpm). The number of words read incorrectly or omitted was recorded. The set of nine test cards with low contrast letters was used first, presented in pseudo-random order. Following a five minute rest, the process was repeated for the high contrast test cards. 

The effects of line space, word space and any interactions were analyzed using a two-way repeated measures ANOVA, with the assumptions of normality and sphericity assessed using Levene’s Test of homogeneity and Mauchly’s Test of sphericity, respectively. A significance level of 0.05 was chosen. Post-hoc analyses were completed using Tukey’s Honestly Significant Difference (HSD) test. 

### Ethics Statement

All experimental and consent procedures were approved by The Aston University Ethics Committee, the NHS South West 2 Research Ethics Committee, and the local ethics committee of the Gloucestershire Hospitals NHS Foundation Trust prior to the commencement of the study. Following verbal and written explanations of the study, written consent was received from each participant. The study adhered to the tenets of the Declaration of Helsinki.

## Results

There were 15 females and 9 males (mean age, 81.4 yrs; sd, 6.9 yrs), with binocular distance visual acuity ranging from 0.3 to 0.74 logMAR (mean, 0.45; sd, 0.13) on the EDTRS chart with best correction. All were receiving Lucentis therapy for wet macular disease to either one (n = 20) or both eyes (n = 4). All were native English speakers and, with the exception of non-central lenticular opacities, none had any ocular comorbidity. 

Critical print size (CPS) was measured in all individuals using both standard (high contrast, 87.5%) and non-standard (low contrast, 17.5%) MNREAD test charts. This was done to ensure each individual had sufficient reading acuity to complete the full study on the effects of text spacing (i.e. no higher than 0.8 logMAR for both the high- and low-contrast MNREAD charts). Participant details (macular condition, distance acuity and CPS) are reported in Table 1. For the high contrast MNREAD chart, CPS varied from 0.2 to 0.8 logMAR (mean, 0.42; sd, 0.17); for the low contrast chart, CPS varied from 0.4 to 0.8 logMAR (mean, 0.63; sd, 0.15).

**Table 1:** Characteristics of study participants.

**No.**	**Age (yrs)**	**Condition**	**DVA (logMAR)**	**CPS (logMAR)**
1	84	R, disciform scarring; L, occult CNV	R, 1.36; L, 0.54; OU, 0.54	HC, 0.6; LC, 0.8
2	79	R, classic CNV; L, classic CNV	R, 0.58; L, 0.78; OU, 0.54	HC, 0.6; LC, 0.8
3	89	R, occult CNV; L, occult CNV	R, 0.34; L, 0.64; OU, 0.3	HC, 0.4; LC, 0.4
4	82	R, dry AMD; L, classic CNV	R, 0.54; L, 0.68; OU, 0.5	HC, 0.5; LC, 0.7
5	84	R, classic CNV; L, dry AMD	R, 0.52; L, 0.32; OU, 0.3	HC, 0.3; LC, 0.5
6	90	R, classic CNV; L, geographic atrophy	R, 0.44; L, CF; OU, 0.44	HC, 0.4; LC, 0.6
7	89	R, disciform scarring; L, occult CNV	R, HM; L, 0.38; OU, 0.38	HC, 0.3; LC, 0.4
8	63	R, classic CNV; L, dry AMD	R, 0.96; L, 0.34; OU, 0.34	HC, 0.2; LC, 0.6
9	83	R, disciform scarring; L, classic CNV	R, 1.64; L, 0.48; OU, 0.48	HC, 0.7; LC, 0.8
10	69	R, dry AMD; L, polypoidal lesion	R, 0.54; L, 0.68; OU, 0.52	HC, 0.5; LC, 0.8
11	85	R, occult CNV; L, dry AMD	R, 0.54; L, 0.56; OU, 0.52	HC, 0.5; LC, 0.6
12	86	R, disciform scarring; L, occult CNV	R, 1.34; L, 0.74; OU, 0.74	HC, 0.8; LC, 0.8
13	87	R, dry AMD; L, classic CNV	R, 0.34; L, 0.62; OU, 0.32	HC, 0.2; LC, 0.4
14	76	R, occult CNV; L, occult CNV	R, 0.42; L, 0.96; OU, 0.44	HC, 0.5; LC, 0.8
15	80	R, classic CNV; L, disciform scarring	R, 0.3; L, 1.36; OU, 0.3	HC, 0.4; LC, 0.5
16	75	R, occult CNV; L, dry AMD	R, 0.32; L, 0.36; OU, 0.32	HC, 0.2; LC, 0.5
17	87	R, classic CNV; L, occult CNV	R, 0.3; L, 0.44; OU, 0.32	HC, 0.3; LC, 0.5
18	82	R, occult CNV; L, disciform scarring	R, 0.68; L, CF; OU, 0.68	HC, 0.3; LC, 0.8
19	80	R, dry AMD; L, classic CNV	R, 0.46; L, 0.72; OU, 0.42	HC, 0.4; LC, 0.6
20	85	R, classic CNV; L, disciform scarring	R, 0.70; L, CF; OU, 0.72	HC, 0.7; LC, 0.8
21	90	R, disciform scarring; L, occult CNV	R, 1.36; L, 0.36; OU, 0.36	HC, 0.3; LC, 0.5
22	83	R, dry AMD; L, classic CNV	R, 0.32; L, 0.64; OU, 0.34	HC, 0.2; LC, 0.5
23	72	R, classic CNV; L, dry AMD	R, 0.70; L, 0.42; OU, 0.48	HC, 0.3; LC, 0.6
24	74	R, occult CNV; L, disciform scarring	R, 0.43; L, 1.36; OU, 0.4	HC, 0.5; LC, 0.8

A binocular add of +2.50 DS was given to each participant to correct for a viewing distance of 40 cms. Monocular (R and L) and binocular (OU) Distance Visual Acuity (DVA) is shown for each participant (HM, Hand Movement; CF, Counting Fingers). Critical Print Size (CPS) is shown for both High Contrast (HC, 87.5% Michelson contrast) and Low Contrast (LC, 17.5%) MNREAD test charts. See text for further details.

Following the CPS measures, the time taken to read short passages of text that varied in word and/or line spacing was measured for each screened participant using both the high- (87.5%) and low-contrast (17.5%) experimental test reading cards. For each passage of text, reading speed was computed as the number of correctly-read words per minute (wpm).

### Effect of word spacing

**Figure 1:**
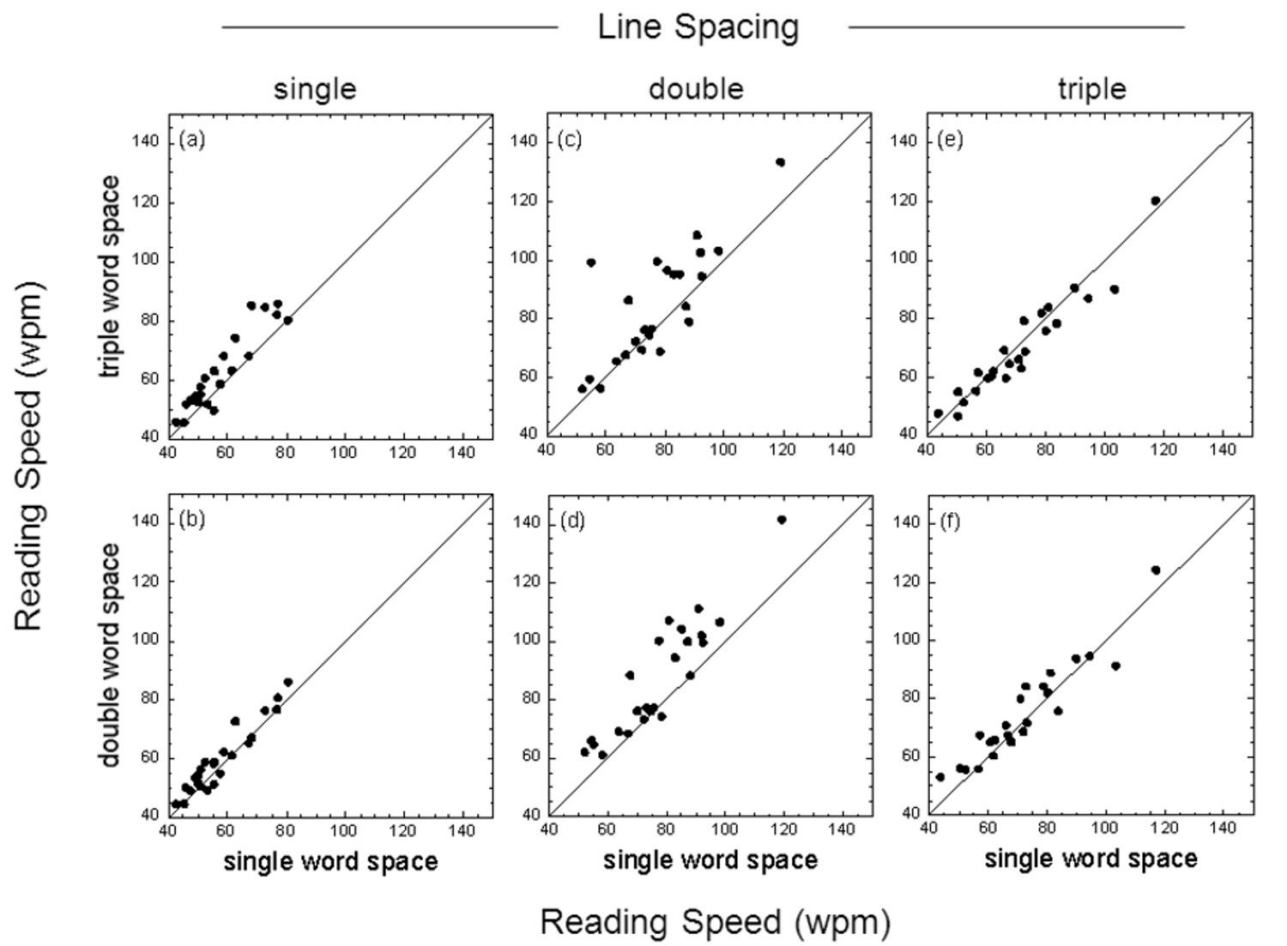
Effect of word spacing with low contrast text. Reading speed (number of correctly-read words per minute, wpm) for single- versus double-character word spacing (bottom panels) and single- versus triple-character word spacing (top panels) for text passages with single, double or triple line spacing. Results shown are for reading text with a Michelson letter contrast of 17.5%.The individual data points in each panel show the results for each participant; the diagonal line in each panel is the ‘line of no effect’.

Figure 1 shows the reading speeds for both single- versus double-character word spacing (bottom panels) and single- versus triple-character word spacing (top panels) for text passages with single, double or triple line spacing. The results shown are for the low contrast test cards. The individual data points in each panel show the results for individual participants, while the diagonal line in each panel is the ‘line of no effect’ (i.e. same performance for both word spacings). Note that in several conditions the data are clustered around this diagonal. However, the data lie predominantly above the diagonal in two conditions (panels 1c and 1d), indicating that reading speed increased with increased word spacing: with double line spacing, mean reading speed (n = 24) increased by 9.9 wpm when word space was increased from one to two characters (panel 1d, p < 0.001), and by 7.1 wpm when word space was increased from one to three characters (panel 1c, p < 0.01) (single-character mean, 77.2 se 3.2; double-character mean, 87.1 se 4.1; triple-character mean, 84.3 se 3.9). Full statistical analyses are reported below on the group-mean reading speeds.

**Figure 2:**
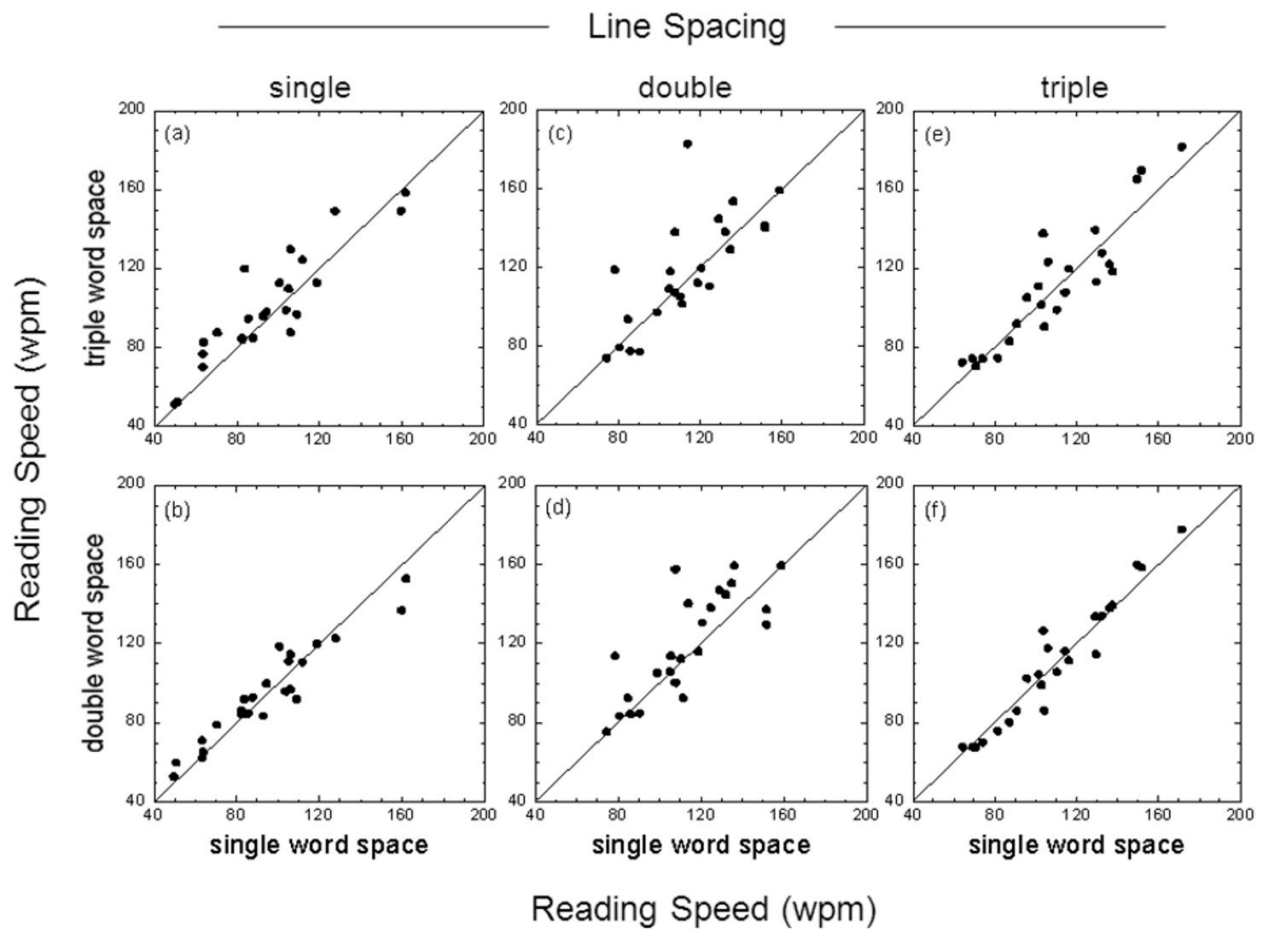
Effect of word spacing with high contrast text. Reading speed (wpm) for single- versus double-character word spacing (bottom panels) and single- versus triple-character word spacing (top panels) for text passages with single, double or triple line spacing. Results shown are for reading text with a letter contrast of 87.5%.The individual data points in each panel show the results for each participant; the diagonal line in each panel is the ‘line of no effect’.

Figure 2 shows the reading speed measures obtained using high contrast test cards. Reading speeds are shown for both single- versus double-character word spacing (bottom panels) and single- versus triple-character word spacing (top panels) for text passages with single, double or triple line spacing. Note that, with high contrast text, the data are clustered around the diagonal in each condition, though the reading speed of a few individuals was substantially increased (> 20 wpm) with double or triple word spacing when combined with double line spacing (Figure 2c, d). Note also that the contrast of the text itself had a large effect: increasing text contrast from 17.5% to 87.5% increased reading speed by a factor of approximately 1.5 (averaged across all conditions). 

### Effect of line spacing

**Figure 3:**
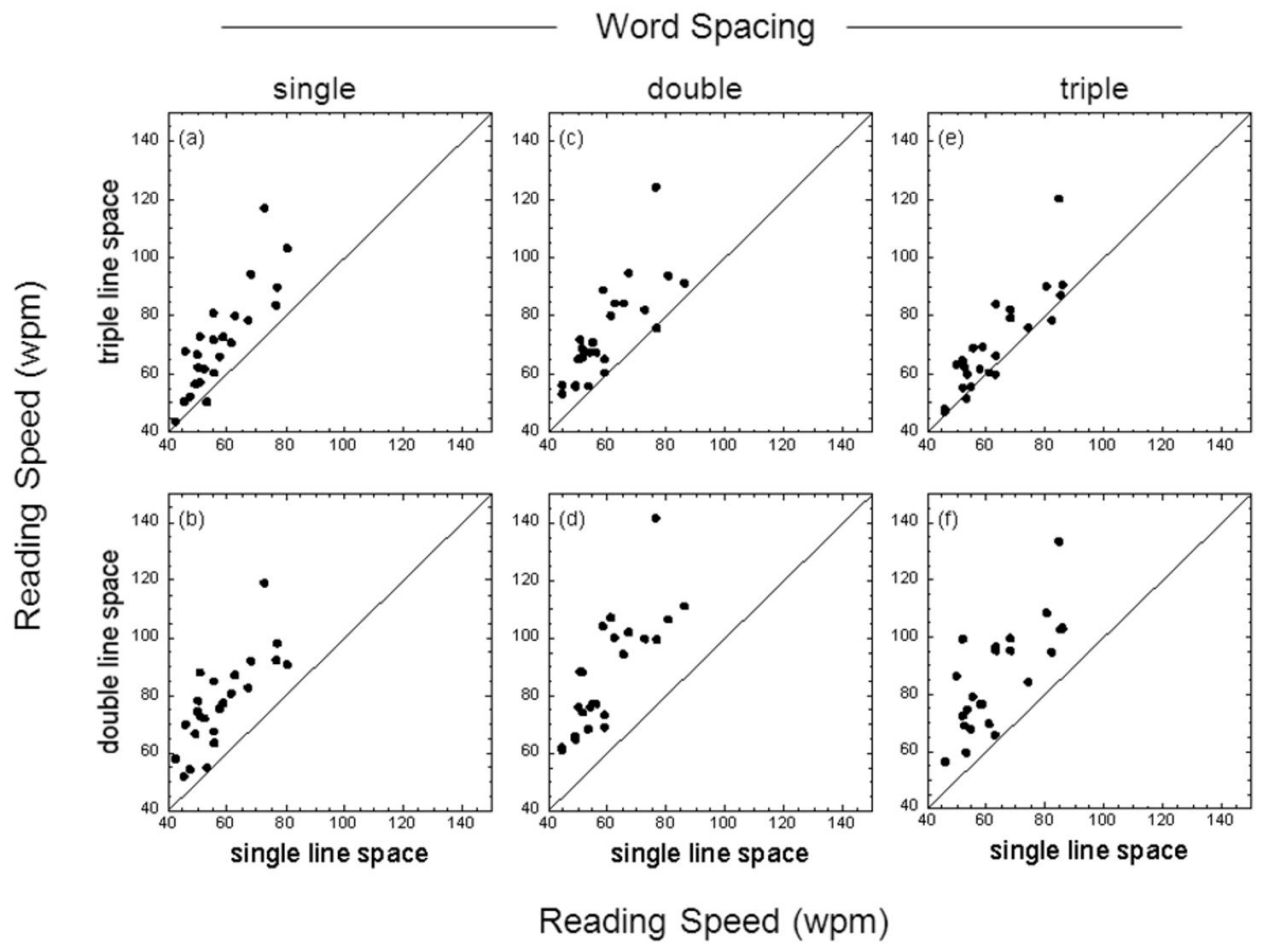
Effect of line spacing with low contrast text. Reading speed (wpm) for single- versus double-line spacing (bottom panels) and single- versus triple-line spacing (top panels) for text passages with single-, double- or triple-character word spacing. Results shown are for text with a Michelson letter contrast of 17.5%. The individual data points in each panel show the results for each participant; the diagonal line in each panel is the ‘line of no effect’.

**Figure 4:**
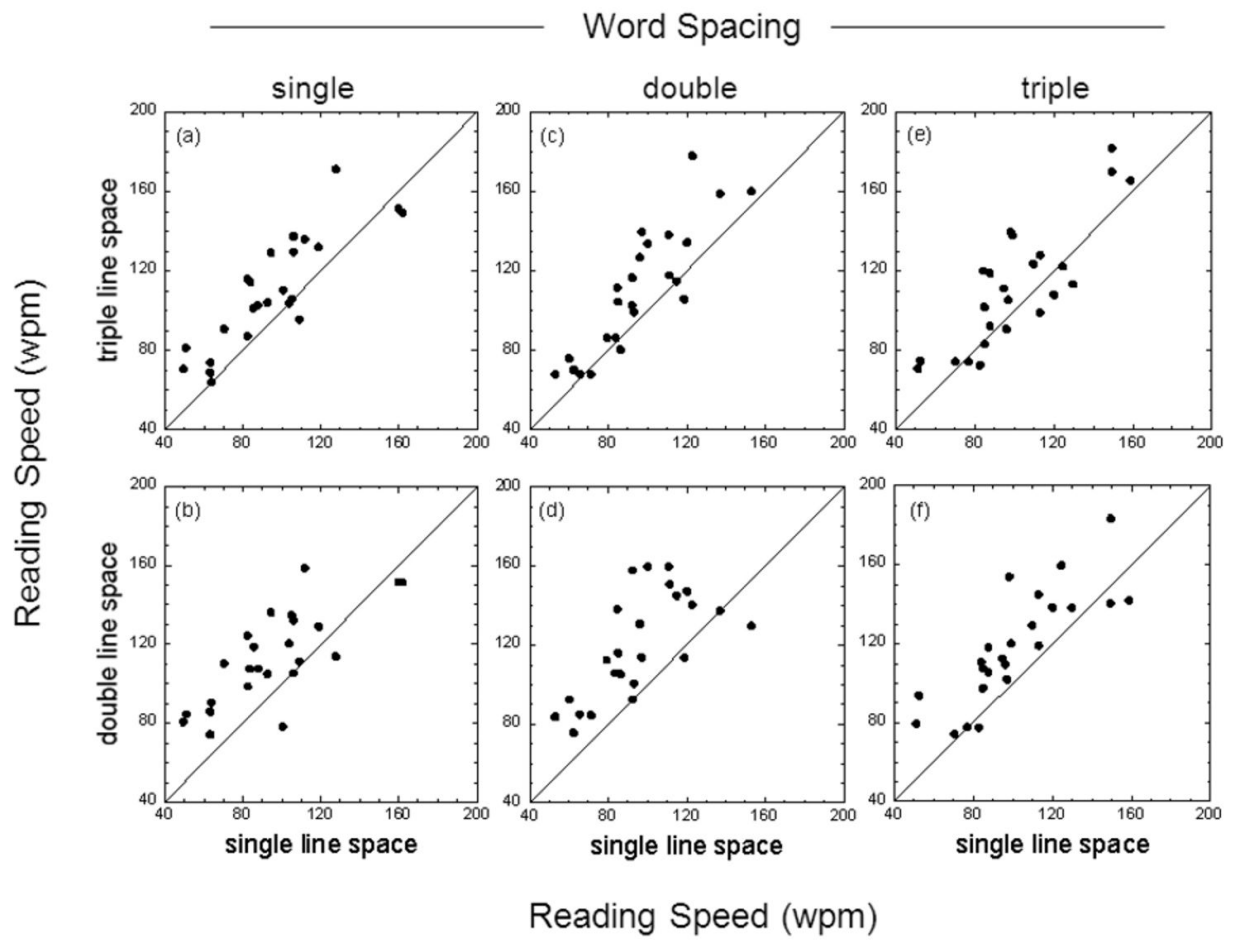
Effect of line spacing with high contrast text. Reading speed (wpm) for single- versus double-line spacing (bottom panels) and single- versus triple-line spacing (top panels) for text passages with single-, double- or triple-character word spacing. Results shown are for text with a letter contrast of 87.5%. The individual data points in each panel show the results for each participant; the diagonal line in each panel is the ‘line of no effect’.

The reading speed data reported above were replotted to highlight the influence of line spacing. Figure 3 shows, for low contrast test cards, reading speeds for both single- versus double-line spacing (bottom panels) and single- versus triple-line spacing (top panels) for text passages with single-, double- or triple-character word spacing. Note that for each word spacing used the data lie above or predominantly above the line of no effect, indicating that both double (p < 0.001) and triple line spacing (p < 0.001) yielded significantly greater reading speeds than single line spacing. A similar pattern of results was obtained for the high contrast test cards (see Figure 4). 

### Group-mean reading speeds

**Figure 5:**
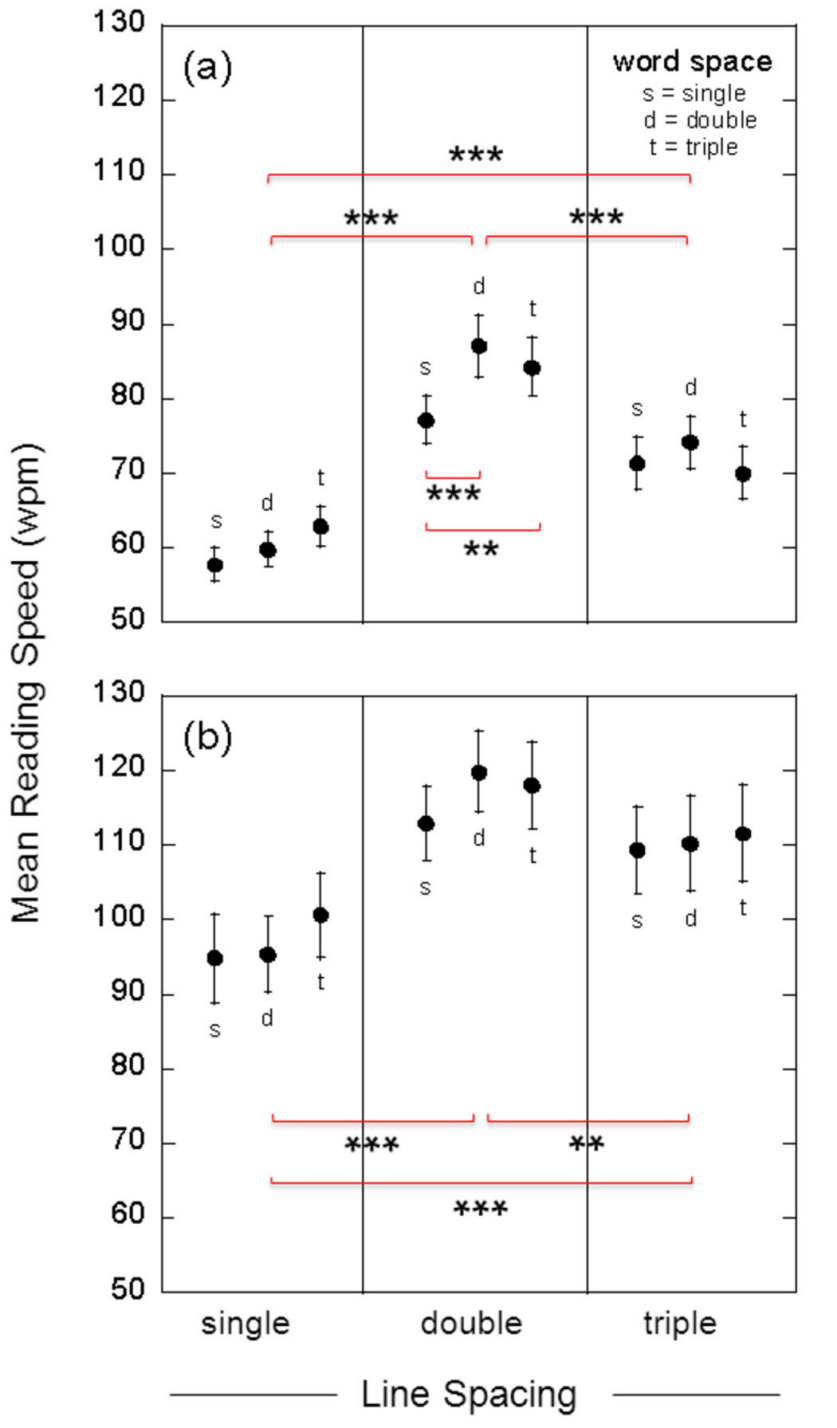
Group-mean reading speeds. Group mean (n = 24) reading speeds (words per minute, wpm) for single, double and triple line spacing. Results are shown for both low contrast text (17.5%, panel a) and high contrast text (87.5%, panel b). For each line spacing, results are shown for single- (s), double- (d) and triple-character (t) word spacing. Error bars show +/- one standard error. Statistical analysis of the data, using a two-way repeated measures ANOVA, is reported in the text. The horizontal red brackets indicate significant differences between conditions, as determined using post-hoc comparisons with Tukey’s HSD test. Shown are the significant differences in mean reading speed between line spacing conditions (averaged across word spacing), and the significant differences between word spacing conditions for low contrast text displayed with double line space (**, p < 0.01; ***, p < 0.001).

Figure 5 shows, for both low (a) and high contrast text (b), group mean (n = 24) reading speeds (wpm) for single, double and triple line spacing. For each line spacing, results are shown for single (s), double (d) and triple (t) word spacing. Averaged across all conditions, the mean reading speed (wpm) obtained with low contrast test cards was 71.6 (se, 1.3), while that obtained with high contrast cards was 108.1 (se, 2.0).

For reading speeds measured with low contrast text (Figure 5a), a two-way repeated measures ANOVA revealed main effects of line space [F(2,46) = 93.71, p < 0.001, ŋ^2^_p_ = 0.2601] and word space on reading speed [F(2,46) = 20.15, p < 0.001, ŋ^2^_p_ = 0.0176]. A significant interaction between line space and word space was also observed [F(4,92) = 9.33, p < 0.001, ŋ^2^_p_ = 0.0154]. The generalized eta-squared measure of effect size (ŋ^2^_p_) was 14.8 times greater for line space than word space. Note that Levene’s Test for homogeneity of variance was not significant (F = 1.84, p = 0.07), indicating that the assumption of equal variances was met. Note also that Mauchly’s Test of sphericity was significant (p < 0.05) for the main effects of line space and the interaction, and as such all reported p values are the Greenhouse-Geisser corrected values. Post-hoc comparisons using the Tukey HSD test indicated that, averaged across the results for different word spacings, the mean reading speed (wpm) measured with either double (82.9, se 2.2) or triple line spacing (71.9, se 2.0) was significantly greater than that measured with single line spacing (60.1, se 1.4; p < 0.001 in both cases). Additionally, reading speed was significantly greater with double than triple line spacing (p < 0.001). Post-hoc comparisons also indicated that, with double line spacing, the reading speed obtained with either double (87.1, se 4.1) or triple word spacing (84.3, se 3.9) was significantly greater than that for single word spacing (77.2, se 3.2; p < 0.001 and p < 0.01, respectively). In Figure 5, these significant differences between conditions are depicted using horizontal red brackets. 

A similar pattern of results was obtained for reading speeds with high contrast text, though the effects were not as pronounced (Figure 5b). A two-way repeated measures ANOVA revealed main effects of both line space [F(2,46) = 22.35, p < 0.001, ŋ^2^_p_ = 0.0841] and word space on reading speed [F(2,46) = 3.98, p < 0.03, ŋ^2^_p_ = 0.0043]. For measures obtained with high contrast text, the main effects were not qualified by an interaction between line and word space [F(4,92) = 1.50, p = 0.23, ŋ^2^_p_ = 0.0029]. The measure of effect size (ŋ^2^_p_) was 19.6 times greater for line space than word space. Again, Levene’s Test for homogeneity of variance was not significant (F = 0.39, p = 0.92). Mauchly’s Test of sphericity was significant (p < 0.05) for the interaction, and therefore the reported p values are the Greenhouse-Geisser corrected values. Post-hoc comparisons indicated that, averaged across the results for different word spacings, the mean reading speed (wpm) measured with either double (116.9, se 3.1) or triple line spacing (110.4, se 3.5) was significantly greater than that measured single line spacing (96.9, 3.2 se; p < 0.001 in both cases). As with the results for low contrast text (Figure 5a), mean reading speed with high contrast text was significantly greater for double than triple line spacing (p < 0.01, Figure 5b).

### Group-mean reading errors

**Figure 6:**
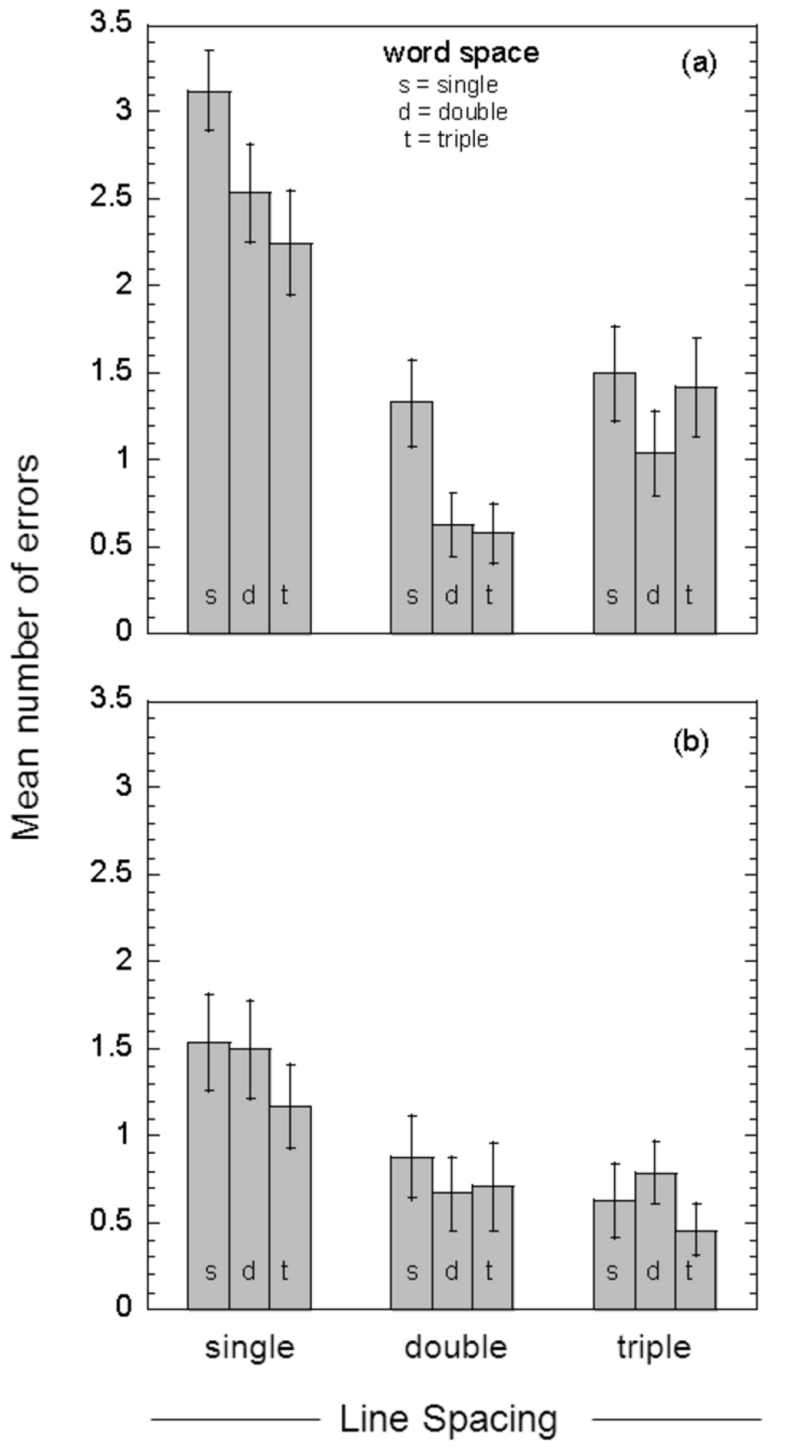
Group-mean reading errors. Group-mean reading errors (number of incorrectly-read or omitted words) for both low contrast text (17.5% contrast, panel a) and high contrast text (87.5% contrast, panel b), plotted as a function of line spacing. For each line spacing, results are shown for single- (s), double- (d) and triple-character (t) word spacing. Error bars show +/- one standard error.

For each condition used, a small number of words (out of a total of 51 words per condition) was read incorrectly or omitted. These errors were recorded and are shown as group-mean values in Figure 6 for both low (a) and high contrast text (b). For each line spacing, results are shown for single (s), double (d) and triple (t) word spacing. Averaged across different word spacings, the number of errors recorded when reading low contrast text was 2.6 (se, 0.2), 0.9 (se, 0.2) and 1.3 (se, 0.2) for single, double and triple line space, respectively (Figure 6a). And for high contrast text, the number of errors was 1.4 (se, 0.2), 0.8 (se, 0.1) and 0.6 (se, 0.1) for single, double and triple line space, respectively (Figure 6b). Averaged across all conditions, the mean number of errors made when reading low contrast text was 1.6 (se, 0.1), while the mean number obtained with high contrast text was 0.9 (se, 0.1). Note that the general findings reported above for reading speed (Figure 5) are reflected in the number of errors recorded for each condition: slower reading speeds were generally associated with a higher number of reading errors. 

## Discussion

**Figure 7:**
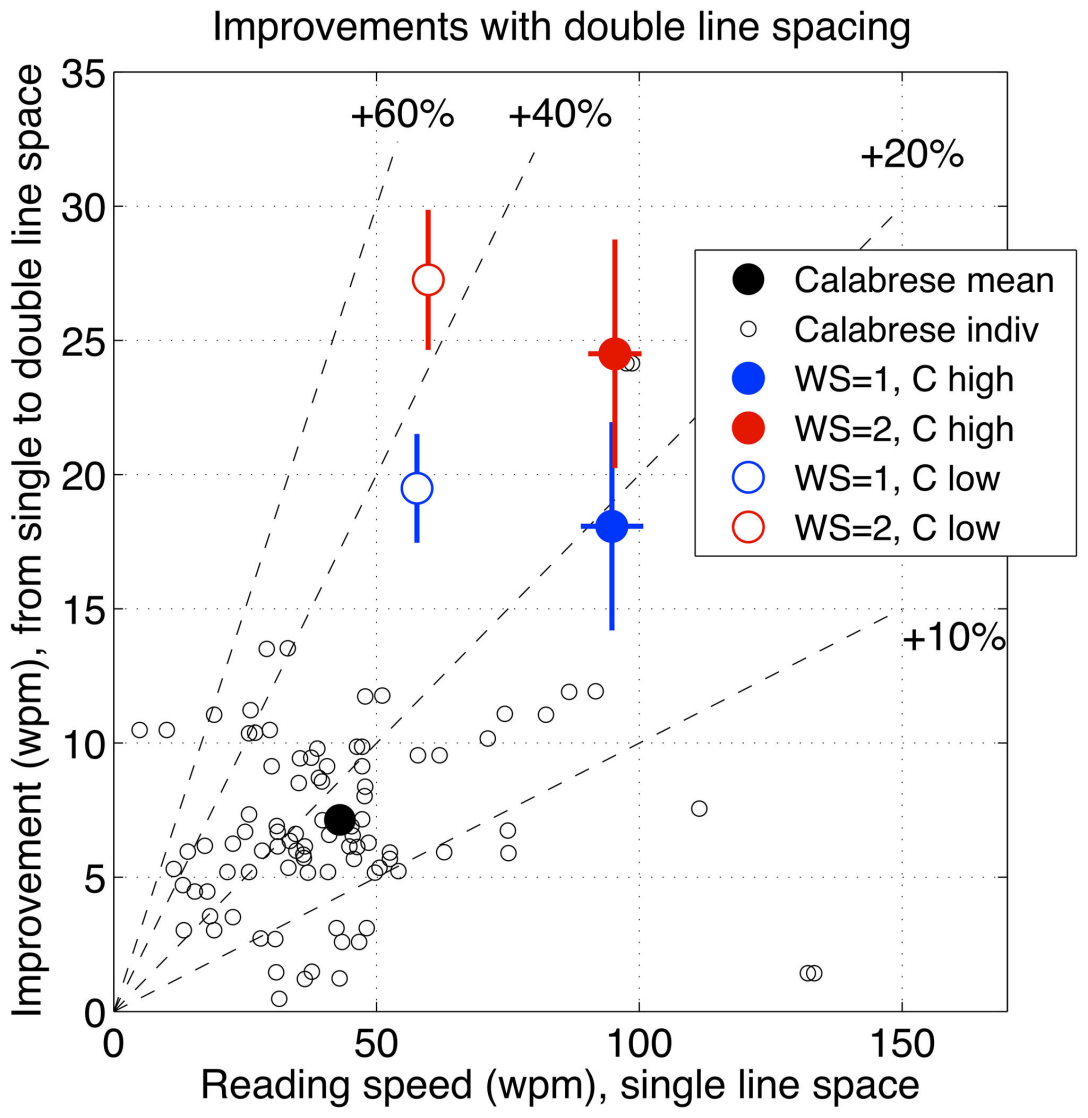
Comparison of reading studies in ARMD. Improvements in mean reading speed of ARMD patients produced by double line spacing, from the present study and Calabrese et al. [45]. Improvement is defined as reading speed with double line spacing minus reading speed with single line spacing, in wpm. Abscissa plots baseline reading speed with single line spacing. Dashed lines represent 10%, 20%, 40% and 60% improvement, as marked. Large circles are mean reading speeds (wpm): black circle, Calabrese et al. [45]; red and blue circles, means ± 1 se from the present study, with single or double word spacing (WS), and low or high contrast (C). Small circles: individual observers from Calabrese et al. [45]. See Discussion, and Table 2.

In this study we assessed the effects of word and/or line spacing on the ability of individuals with age-related macular degeneration to read short passages of text (three sentences, 51 words) that were printed with either high (87.5%) or low contrast (17.5%) letters. Low contrast text was used to enhance test sensitivity and avoid potential ceiling effects, and also to mimic a possible reduction in letter contrast with light scatter from media opacities. We employed whole sentences, binocular viewing and free saccadic eye movements for all measures because we wanted to model as closely as possible the natural reading process. Our results provide evidence that enhanced text spacing significantly increases reading speed and reduces the number of reading errors in individuals with macular disease. For both low and high contrast text, the fastest reading speeds we measured were for passages of text with double line and double word spacing (Figure 5). In comparison with standard single spacing, double word/line spacing increased reading speed by approximately 26% (p < 0.001) with high contrast text, and by 46% (p < 0.001) with low contrast text (Table 2, Figure 7). In addition, double word/line spacing more than halved the number of reading errors obtained with standard single spaced text (Figure 6).

**Table 2:** Comparison of reading studies in ARMD.

**Data source**	**Measure**	**Speed, wpm 1-line space**	**Speed, wpm 2-line space**	**Improvement, wpm**	**Improvement, %**
Calabrese et al 2010	Mean	43.02	50.17	7.14	16.60
*(N=90 eyes, 61 Ss)*	Median	38.20	45.75	6.32	16.55
Chung et al 2008, RSVP	Mean	43.10	62.59	19.49	45.22
*(N=4 Ss)*	Median	41.09	67.98	22.47	54.68
WS=1, high contrast	Mean	94.80	112.88	18.08	19.07
*(N=24 Ss, our data)*	Median	93.23	110.67	21.11	22.64
WS=2, high contrast	Mean	95.34	119.84	24.50	25.70
	Median	92.31	114.83	24.68	26.74
WS=1, low contrast	Mean	57.71	77.20	19.48	33.76
	Median	55.23	76.41	18.88	34.18
WS=2, low contrast	Mean	59.82	87.07	27.25	45.56
	Median	57.27	82.72	23.99	41.90

Summary of improved reading speeds with double line-spacing in three studies of ARMD patients. WS = word spacing. Ss = subjects. Improvement in wpm, for a given observer, is reading speed with double line spacing minus reading speed with single line spacing. The column ‘*Improvement,wpm’* shows the mean or median improvement. For the median, this is not in general equal to the difference between the two median speeds. The column ‘*Improvement, %’* is the mean (or median) improvement as a percentage of the mean (or median) reading speed with single-line spacing.

Line spacing was more important than word spacing. Although a repeated-measures ANOVA revealed main effects of both line and word spacing, the measure of effect size (ŋ^2^_p_) was approximately fifteen to twenty times greater for line spacing than word spacing. For both low and high contrast text, the mean reading speed obtained with either double or triple line spacing was significantly greater than that obtained with single line spacing (results averaged across different word spacings). Additionally, the mean reading speed obtained with double line spacing was significantly greater than that obtained with triple line spacing. The benefits of enhanced word spacing were less clear. While double and triple word spacing were helpful for some individuals (see raw data in Figures 1-4), post-hoc comparisons between different word spacing conditions generally failed to reach statistical significance. For low contrast text, however, post-hoc comparisons indicated that both double and triple word spacing significantly augmented the benefits of double line spacing (Figure 5a). 

The benefits we did observe from enhanced word spacing alone are consistent with reports that increased inter-word spacing facilitates shorter fixation durations [49-51], while reduced inter-word spacing is detrimental to reading speed [46-48]. Slattery and Rayner [49] have recently argued that these inter-word spacing effects may reflect improved word segmentation processes, whereby low spatial frequency information from widely spaced text serves to guide saccade planning to individual word units. 

Unlike the studies on inter-word spacing, previous studies on the effects of inter-line spacing have yielded mixed results that deserve careful consideration. With whole sentences, increased interline spacing was reported to yield either a small (though significant) increase in mean reading speed of macular disease patients [45] or no mean change at all [44]. The first of these studies is presumably more reliable than the second because it had a much larger sample size (N = 90 eyes, 61 observers *versus* N = 8 observers). The data from this study [45] are re-plotted in Figure 7. Improvements for individual observers (small open circles) produced by double line spacing ranged from below 10% to more than 40%. For comparison, results from four of our main conditions are re-plotted here in the same format (using large red and blue circles). Importantly, we can now see that for the text condition most comparable to that of Calabrese et al. [45] (word spacing =1, with high contrast; filled blue circle) our improvement in mean speed (19%) was similar to theirs (17%), despite a large difference in baseline reading speeds (see also Table 2). We agree with Calabrese et al. that these are modest improvements. But Figure 7 reinforces what we have already seen in Figure 5 – that doubling word spacing (red symbols) enhanced the effect of double line spacing, such that the improvement in mean speed increased to 26% with high contrast text and 46% with low contrast text (Table 2). Thus double-line and double-word spacing was particularly helpful to these ARMD patients when text contrast was low – the *combined* benefit of double line and word spacing over single line and word spacing was 51%. Of course this is not a recommendation to use low contrast text, because actual reading speed is always higher at high contrast (Table 2, Figure 5). However, should the contrast of the text viewed by individuals with macular disease be reduced because of intraocular light scatter or poor viewing conditions, it is clear that a doubling of both word and line spacing would benefit their reading performance. 

We noted above that Chung et al. [44] found no mean advantage for increased line spacing in a group of eight ARMD patients reading whole sentences. In light of the findings discussed above, it seems likely that the modest effect at high contrast (less than 20%) was lost in the noise of a relatively small group of patients. Using a different procedure (rapid serial presentation of single words, RSVP), we note that Chung et al. [44] did find a consistent improvement for double versus single line spacing (Table 2). Although this was again a small sample (N=4), it is interesting that the improvement in mean speed with RSVP (about 45%) was similar to our results at low contrast.

Why should double line/word spacing be especially effective in enhancing reading performance? It seems clear that minimizing the deleterious effects of visual crowding must be at least part of the answer. In addition, we suspect that enhanced line spacing may also help to minimize the number of unnecessary eye movements made by visually impaired persons when saccading from the end of one line to the beginning of the line below. Unfortunately, we do not have direct evidence for this. However, this explanation is consistent with the hypothesis that additional blank spaces may improve word segmentation [49]. Moreover, anecdotal observations by clinicians (including two of the authors) suggest that, with single line spacing, individuals with macular disease often begin to re-read the same line, apparently uncertain of which line was the next line down. A line of text in relative isolation presumably provides a more powerful cue for directing eye movements [58].

Macular disease proves to be devastating for many people. There are various reasons why this is so but principal among them is the inability of individuals with poor central vision to read efficiently [59]. The latter robs such individuals of their independence, thereby reducing their quality of life [59-61]. The results of this study suggest that enhanced text spacing reduces the detrimental effects of peripheral visual crowding, yielding a significant increase in reading speed and a reduction in the number of reading errors. We recommend that individuals with macular disease should, whenever possible, employ double line spacing and double-character word spacing to maximize their reading efficiency. Today, such changes are easily implemented with many modern handheld reading tablets. Reading tablets also have the advantage of being able to display text at or near maximum contrast.

The challenge remains, of course, to improve reading efficiency with standard single-spaced text that is not amenable to manipulation, including most printed novels, magazines and medicine labels. But here too, recent research provides some hope. For example, psychophysical work on populations of amblyopes and normally-sighted observers [27,62,63] suggests that crowding may be reduced following perceptual training on flanked letter identification tasks. Importantly, the results show that the learning effects may be long lasting and not restricted to any particular age group. These findings are supported by recent theoretical arguments on the reduction of crowding through learning [64]. Such learning is considered to be the perceptual manifestation of cortical visual plasticity, which – it is becoming increasingly clear – can be activated in later life [63]. We speculate that the use of enhanced text spacing in conjunction with perceptual training to reduce further the effects of crowding might provide a useful clinical protocol for maximizing reading performance in macular disease.
